# Specific DNA mini-barcoding for identification of *Gekko gecko* and its products

**DOI:** 10.1186/s13020-020-00382-2

**Published:** 2020-09-29

**Authors:** Yanyan Su, Dandan Ding, Mengjie Yao, Lan Wu, Gangqiang Dong, Dong Zhang, Shilin Chen, Li Xiang

**Affiliations:** 1 Institute of Chinese Materia Medica, China Academy of Chinese Medical Sciences, Beijing, 100700 China; 2 Amway (China) Botanical Research and Development Center, Wuxi, 214145 China; 3 College of Agricultural and Environmental Sciences, University of California, Davis, CA 95616 USA

**Keywords:** *Gekko gecko*, specific primers, DNA mini-barcoding, Identification

## Abstract

**Background:**

The dry body of the Tokay Gecko (*Gekko gecko*) is the source of a valuable traditional Chinese medicine, it is therefore listed as a Class II protected animal species in China. Due to increasing market demand and a declining supply of the species, a considerable number of adulterants have emerged in the market. Thus, it is necessary to establish an accurate and rapid method of identification for distinguishing *G. gecko* from its adulterants and for separating it from highly processed products.

**Methods:**

A total of 274 *COI* sequences were analyzed by using MEGA 5.0 software. Several specific primers were designed to amplify mini-barcode regions and identify *G. gecko* from its counterfeits and products.

**Results:**

274 *COI* sequences of *G. gecko* and 15 adulterants species were analyzed. *G. gecko* could be distinguished from its adulterants through BLAST analysis, intra- and inter-specific distance analyses, and an NJ tree based on *COI* sequences. Two pairs of specific primers designed for this study, COISF2/COISR2 and COISF3/COISR3, amplified 200- and 133-bp fragments of the *COI* region, respectively, both of which were suitable for the identification of *G. gecko* and its adulterants. Furthermore, COISF3/COISR3 detected *G. gecko* in 15 batches of products.

**Conclusion:**

Therefore, the specific DNA mini-barcoding method developed here may be a powerful tool for the identification of *G. gecko* and counterfeits, and may also be used to distinguish *G. gecko* from its highly processed by-products.

## Background

As one of the rarest Chinese medicinal materials, the dry body of Tokay Gecko *(G. gecko* Linnaeus), has been used for two thousand years and is known for its remarkably therapeutic effect on kidney deficiency [1, 2]. Previous studies have found that *G. gecko* also has notable effects in relieving asthma, strengthening the immune system, and treating tumors [3–5]. Today it is widely used in many functional foods and Chinese patent medicines, such as tinctures, Renshen Gejie Powder, Gejie Dingchuan Capsule, Gejie Dingchuan Pill, and Shenge Pingchuan Capsule [6, 7]. For decades, the medicinal demand for *G. gecko* has skyrocketed in China. However, due to the destruction of its natural habitats and indiscriminate hunting, the wild *G. gecko* has been classified as a Class II protected animal species in China since 1989 [8]. As a result, the demand for the animal is considerably higher than its supply. Currently, the domestic market relies on imported Tokay Gecko from Southeast Asia and native-cultured *G. gecko* [9]. Increasing market demand and the declining supply of the native *G. gecko* increases its value; consequently, a considerable number of adulterants have emerged in the market, including *Gekko swinhonis*, *Hemidactylus frenatus*, *Laudakia himalayana*, *Eumeces chinensis*, *Batrachuperus pinchonii*, and other common adulterants [10–12]. Besides their very similar appearances, their ambiguous common names have also exacerbated the difficulty in distinguishing *G. gecko* from counterfeits. For example, *G. swinhonis* is commonly known as little Gecko, and *L. himalayana* is known as the Tibet Gecko [13]. The indistinguishable adulterants infiltrate the markets and severely damage public safety. Thus, an efficient identification method needs to be urgently developed.

At present, the most frequently used method of identifying *G. gecko* involves observing its morphological characteristics and conducting microscopic analyses [14–16]; it is not practical for researchers without knowledge of taxonomy to differentiate between incomplete samples and powders based on morphological characteristics [17–19]. No reliable method of identification has been reported because no specific chemical component functioning as a chemical marker has been found. It is unclear what active component of *G. gecko* is used in related functional foods and Chinese patent medicines [20, 21]. Although DNA barcoding technology based on *COI* region has the ability to distinguish *G. gecko* from related species and to identify other animal species successfully [11, 22, 23], this technology cannot be applied to samples with degraded DNA because it is impossible to amplify the *COI* region (> 600 bp) of highly processed materials [24–27].

Therefore, a new method using DNA mini-barcoding based on the *COI* region combined with a specific PCR technique was invented and used in this study to distinguish *G. gecko* from its adulterants and related products.

## Materials and methods

### Materials

**Fig. 1:**
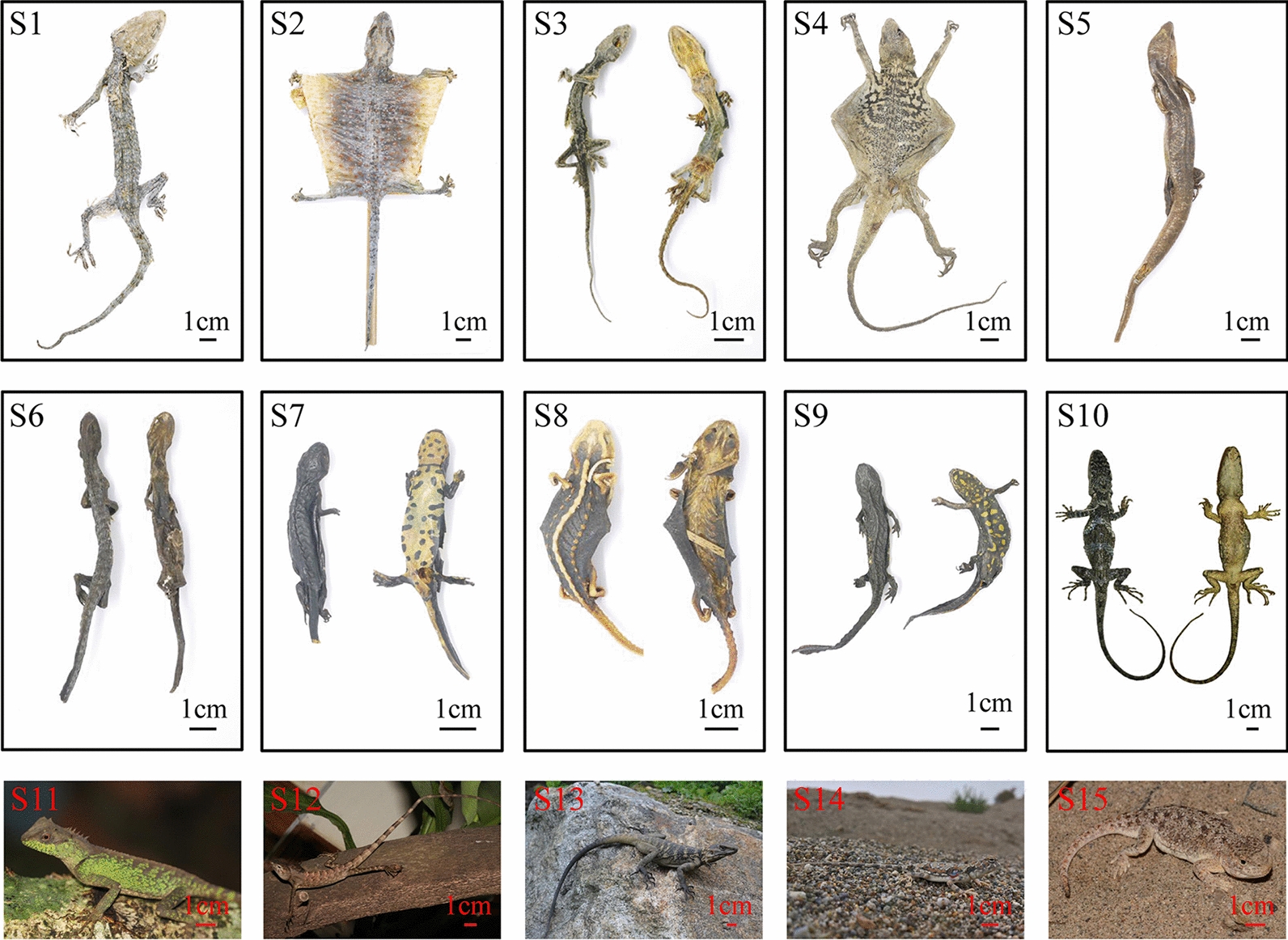
Photos of samples in this study. S1, S2: *G. gecko*, S3: *G. swinhonis*, S4: *L. himalayana*, S5: *E. chinensis*, S6: *B. pinchonii*, S7: *C. orientalis*, S8: *T. shanjing*, S9: *P. chinensis*, S10: *C. kakhienensis*, S11: *A. lepidogaster*, S12: *C. versicolor*, S13: *L. sacra*, S14: *P. axillaris* and S15: *P. theobaldi*

All experiments were performed in accordance with the guidelines for the use and care of animals of the Center for Laboratory Animal Care, China Academy of Chinese Medical Sciences. All experimental protocols were approved by the Research Ethics Committee of Institute of Chinese Materia Medica, China Academy of Chinese Medical Sciences, Beijing, China. A total of 269 samples, including 47 original animals (dry samples from local stores), 19 specimens (dry and dissected samples from Kunming Institute of Zoology, Chinese Academy of Sciences), and 203 medicinal materials belonging to 16 species of *G. gecko* and its adulterants were collected from localities, such as Anguo herb market and Bozhou herb market (see Fig. 1 and Additional file 1: Table S1). A total of 274 *COI* sequences were analyzed, including 269 sequences obtained from this study and five sequences downloaded from GenBank. All the samples of this study were deposited as Voucher at the Institute of Chinese Materia Medica, China Academy of Chinese Medical Sciences, and the obtained 269 *COI* sequences have been submitted to GenBank.

In addition, 15 batches of five kinds of related products were purchased from online stores and drugstores.

### DNA extraction, amplification, and sequencing

In 75% ethanol 10–30 mg of underlying muscles of specimens were rinsed then pulverized using a DNA Extraction Grinder (Sceintz Biotech Co., China). Genomic DNA was extracted using an Animal Genomic DNA Kit (Tiangen Biotech Co., Ltd, Beijing, China). *COI* sequences were amplified with universal primers LCO1490/HCO2198 according to *Standard DNA Barcodes of Chinese Materia Medica in Chinese Pharmacopoeia* [11, 28, 29]. The PCR products were examined using 1.0% agarose gel electrophoresis. The purified PCR products were bi-directionally sequenced using an ABI 3730 XL sequencer (Applied Biosystems Inc.).

### Sequence analysis and primers design

The attained trace files were assembled and trimmed using CodonCode Aligner V 3.0 (CondonCode Co., USA). Then the 269 *COI* sequences were verified in the NCBI database and DNA Barcoding System for Identifying Herbal Medicine (http://www.tcmbarcode.cn/en/). A total of 274 *COI* sequences were analyzed using MEGA 5.0 software, including calculating intra- and inter-specific distances and constructing a neighbor-joining (NJ) tree based on the Kimura 2-Parameter model. Sequentially, two pairs of specific primers of *G. gecko* were designed based on *COI* regions of *G. gecko* (658 bp) using Primer 5.0 software: COISF2: 5′-GCCTCCGCGAGTGTG-3′, COISR2: 5′-CGTGTATTGGGTTATGCTTGG-3′; and COISF3: 5′-AAAATGAAAACCCCAAG-3′, COISR3: 5′-TACGAACGGTCAACAA-3′. A reaction mixture comprising of 12.5 μL 2 × Taq PCR Master Mix (Aidlab Biotechnologies Co., China), 1 μL of each primer (2.5 μM), 1 μL genomic DNA, and 9.5 μL ddH_2_O was prepared. The PCR amplification conditions of COISF2/COISR2 were initially set up as 94 °C for 5 min, followed by 40 cycles of 94 °C for 30 s, 61 °C for 30 s, and 72 °C for 30 s, and a final extension at 72 °C for 10 min. The amplification of COISF3/COISR3 started with 94 °C for 5 min, followed by 40 cycles of 94 °C for 30 s, 50 °C for 30 s, and 72 °C for 30 s, and a final extension temperature at 72 °C for 5 min.

The genomic DNA extracted from *G. gecko* was diluted to a series of concentrations (100 ng/μL, 10 ng/μL, 1 ng/μL, 100 pg/μL, 10 pg/μL, and 1 pg/μL) and then amplified with the specific primers to determine the minimum amplifiable concentration.

### Distinguishing *G. gecko* from related products

Fifteen batches of functional foods and Chinese patent medicines, including three batches of functional foods (Renshen Gejie Powder), twelve batches of Chinese patent medicines (three batches of Gejie Dingchuan Pill, Gejie Dingchuan Capsule, Shenge Pingchuan Capsule, and Gejie Dangshen Syrup each), allegedly containing *G.* g*ecko* were collected from online stores and drugstores. DNA was extracted from all samples in triplicates. Using nuclear separation liquid (100 mM Tris–HCl (pH 8.0), 20 mM EDTA (pH 8.0), 700 mM NaCl, 2% PVP-40, and 0.4% β-mercaptoethanol), 0.5–1.0 g of powdered samples, 5–6 g of pills, and 10 g of syrup were rinsed 1–5 times until the supernatant liquid became lightly colored or colorless [30]. Genomic DNA of these functional foods and Chinese patent medicines was extracted using an Animal Genomic DNA Kit (Tiangen Biotech Co., Ltd, Beijing, China) and amplified with universal primers of *COI* region and specific primers to examine the existence of *G. gecko*. A total of 15 DNA samples were detected with PCR reaction, three samples of each product.

## Results

### Identification of *G. gecko* from adulterants through DNA barcoding

In this study, 274 *COI* sequences belonging to 16 species were analyzed after being verified in the NCBI database and DNA Barcoding System for Identifying Herbal Medicine (see Additional file 1: Table S1). The results are shown in Additional file 1: Table S2. The *COI* sequences for most species were 658 bp long, and *P. axillarisone* was the species with the shortest *COI* sequence (611 bp). The guanine-cytosine (GC) content for all species was lower than 50% except for *H. frenatus* (50.6%); *G. gecko* had a GC content of 48.2% and *B. pinchonii* had the lowest GC content (39.5%). Furthermore, the maximum intra-specific K2P distances between *G. gecko* and 15 adulterants were less than their minimum inter-specific distances, which indicated that every species in this study could be distinguished from other species (see Additional file 1: Table S2). In addition, an NJ tree was constructed using 69 haplotypes of 274 *COI* sequences which belong to 16 species (see Additional file 1: Figure S1). The haplotypes of *G. gecko* clustered into a branch with a bootstrap value of 99, and the haplotypes of counterfeits also formed branches with high bootstrap values. This result correlated with that of the intra- and inter-specific distances analyses suggesting that DNA barcoding could effectively distinguish *G. gecko* from other related species.

### Design and amplification of specific primers

**Fig. 2:**
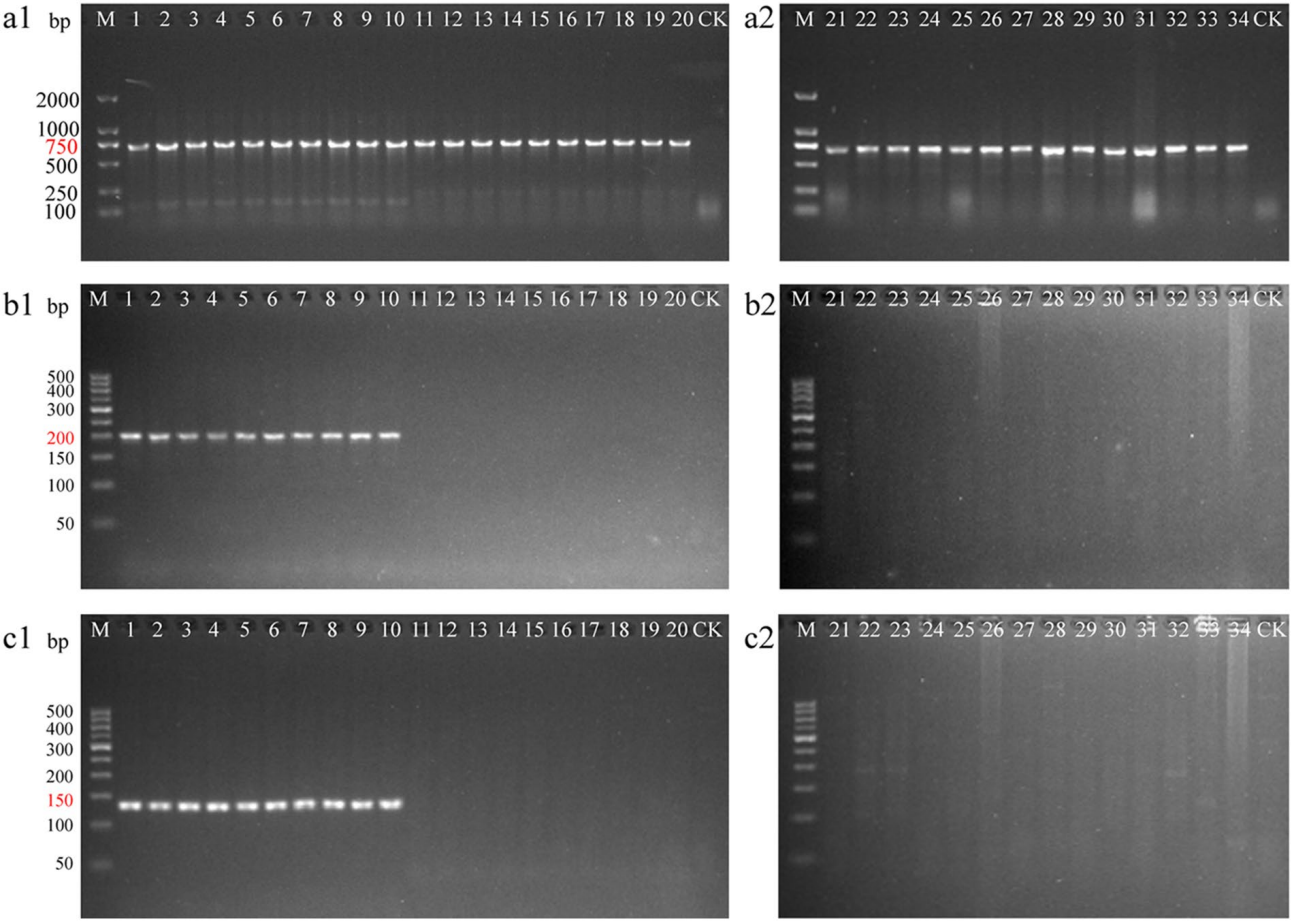
Amplification of partial samples of *Gekko gecko* and its adulterants with universal and specific primers. **a1, a2** Amplification with *COI* universal primer pair LCO1490/HCO2198. **b1, b2** Amplification with *COI* specific primer pair COISF2/COISR2. **c1, c2** Amplification with *COI* specific primer pair COISF3/COISR3. 1-10: *G. gecko*, 11-20: *G. swinhonis*, 21: *H. frenatus*, 22: *A. lepidogaster*, 23: *C. kakhienensis*, 24: *C. versicolor*, 25: *L. himalayana*, 26: *L. sacra*, 27: *P. axillaris*, 28: *P. mystaceus*, 29: *P. theobaldi*, 30: *E. chinensis*, 31: *B. pinchonii*, 32: *C. orientalis*, 33: *P. chinensis*, 34: *T. shanjing*, and CK: negative control

Two specific primers COISF2/COISR2 (COISF2: 5′-GCCTCCGCGAGTGTG-3′, COISR2: 5′-CGTGTATTGGGTTATGCTTGG-3′) and COISF3/COISR3 (COISF3: 5′-AAAATGAAAACCCCAAG-3′, COISR3: 5′-TACGAACGGTCAACAA-3′) amplified 200- and 133-bp fragments of the *COI* region respectively, and were designed for the mini-barcoding of *G. gecko* and to identify related products (Additional file 1: Figures S2 and S3). The universal *COI* primers and two specific primers were used in this study to amplify genomic DNA of *G. gecko* and its 15 adulterants. Using universal primers of *COI*, all samples were successfully amplified and showed markers approximately 750 bp long (Fig. 2a). We found that the template DNA of specimens met the requirement of amplification. Furthermore, when specific primers were used, only *G. gecko* showed expected markers at 200 and 133 bp; however, no other target PCR products could be seen (Fig. 2b, c). The results confirmed that the two primer pairs COISF2/COISR2 and COISF3/COISR3 developed in this study could effectively and distinctly separate *G. gecko* from its adulterants.

### Sensitivity of specific primers

**Fig. 3:**
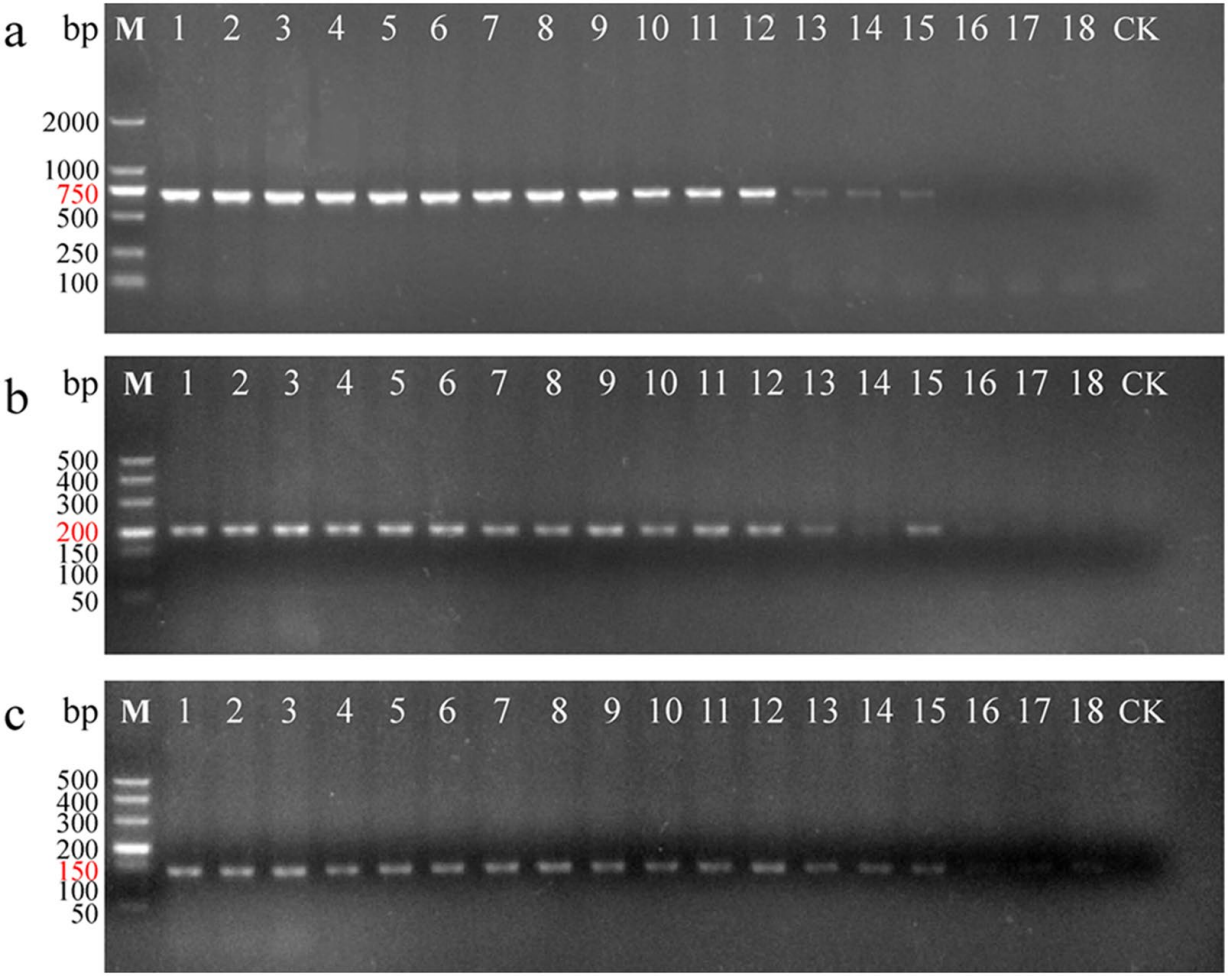
The sensitivity of amplification with universal and specific primers for detection of *Gekko gecko*. **a** Amplification with *COI* universal primer pair LCO1490/HCO2198. **b** Amplification with *COI* specific primer pair COISF2/COISR2. **c** Amplification with *COI* specific primer pair COISF3/COISR3. 1–3: 100 ng/μL, 4–6: 10 ng/μL, 7–9: 1 ng/μL, 10–12: 100 pg/μL, 13–15: 10 pg/μL, 16–18: 1 pg/μL, and CK: negative control

To examine the sensitivity of the two specific primers, template DNA was diluted to a series of concentrations varying from 100 ng/μL to 1 pg/μL. Then, these samples were amplified with three replicates each using universal primers, LCO1490/HCO2198 and the two specific primers, COISF2/COISR2 and COISF3/COISR3 (Fig. 3). The minimum effective concentrations of DNA that could be amplified by these primers were 10 pg/μL. More specifically, when the template DNA concentration was only 1 pg/μL, the band of PCR products with primers COISF3/COISR3 were faintly visible (Fig. 3c). Specific primers COISF3/COISR3 were more sensitive to *G. gecko* than the universal primer and the required minimal template concentration was 1–10 pg**/**μL.

### Distinguishing *G. gecko* from its related products

To verify the utility and practicability of the specific primers, DNA extracted from samples obtained from 15 batches of functional foods were amplified with the universal and specific primers (Table 1). Only *G. gecko* from Renshen Gejie Powder could be successfully amplified with universal primers LCO1490/HCO2198 (Additional file 1: Figures S4a). Using the specific primers COISF2/COISR2, *G. gecko* was successfully amplified from Renshen Gejie Powder and Shenge Pingchuan Capsule (Additional file 1: Figures S4b). For specific primers COISF3/COISR3, the amplification results of five kinds of related products in this study were positive (Additional file 1: Figures S4c). All the PCR products were sequenced to ensure the reliability of amplification, and obtained sequences were verified in the NCBI database and DNA Barcoding System for Identifying Herbal Medicine. The results revealed that all 15 batches of related products in this study contained *G. gecko*. Additionally, it was confirmed that COISF3/COISR3 could be used to distinguish *G. gecko* from its related products.

**Table 1:** Detection results of *Gekko gecko* from related products

Sample no.	Product name	Ingredients number	Repetitions	Amplification results		
				LCO1490/ HCO2198	COISF2/ COISR2	COISF3/ COISR3
RGS1	Renshen Gejie Powder	8	3	Y	Y	Y
RGS2	Renshen Gejie Powder	8	3	Y	Y	Y
RGS3	Renshen Gejie Powder	8	3	Y	Y	Y
SGPC1	Shenge Pingchuan Capsule	4	3	N	Y	Y
SGPC2	Shenge Pingchuan Capsule	4	3	N	Y	Y
SGPC3	Shenge Pingchuan Capsule	4	3	N	Y	Y
GDG1	Gejie Dangshen Syrup	2	3	N	N	Y
GDG2	Gejie Dangshen Syrup	2	3	N	N	Y
GDG3	Gejie Dangshen Syrup	2	3	N	N	Y
DCW1	Gejie Dingchuan Pill	14	3	N	N	Y
DCW2	Gejie Dingchuan Pill	14	3	N	N	Y
DCW3	Gejie Dingchuan Pill	14	3	N	N	Y
DCJN1	Gejie Dingchuan Capsule	14	3	N	N	Y
DCJN2	Gejie Dingchuan Capsule	14	3	N	N	Y
DCJN3	Gejie Dingchuan Capsule	14	3	N	N	Y

Y means target fragments were successfully amplified. N implies no fragments were amplified

## Discussion

Due to over-exploitation of *G. gecko* as a traditional Chinese medicine, it has been listed as a Class II protected animal species. As a result, the demand has always greatly exceeded market supply and various adulterants have emerged in the market, which compromise clinical effects and public safety.

In this study, *G. gecko* and its 15 adulterants were distinguished from each other through DNA barcoding based on *COI* sequences. The results were consistent with a previously reported study that identified *G. gecko* from adulterants using *COI* regions [11, 22]. DNA mini-barcoding and specific-PCR identification are becoming more popular methods for effective species identification [24, 25, 31], especially for specimens with degraded DNA, such as archival specimens, processed foods, functional foods, and Chinese patent medicines [26, 27, 32–35]. For a more sensitive identification of *G. gecko* in 2001, it was distinguished from 14 adulterants through allele-specific diagnostic PCR which amplified 260-bp fragments of the 12S rRNA gene [36]. To distinguish *G. gecko* from 11 adulterants, Gu proposed four specific primer pairs according to the *COI*, *Cytb*, 16S, and 12S rRNA gene sequences of *G. gecko* and amplified 210-, 200-, 345-, and 110-bp fragments, respectively [37]. Jiang applied a specific PCR method to amplify approximately 400-bp fragment of the *COI* region to identify *G. gecko* from seven common adulterants [38]. Although significant efforts have been made by researchers, degraded DNA extracted from *G. gecko*-relevant functional foods and Chinese patent medicines have yet to be tested.

In this study, specific primers COISF2/COISR2 and COISF3/COISR3 were designed to amplify mini-barcode. The primers could amplify 200- and 133-bp *COI* fragments, respectively. The specific primers COISF3/COISR3 can be used not only for the identification of original animal of *G. gecko* from its adulterants, but also for the identification of functional foods and Chinese patent medicines. Similar to other DNA mini-barcoding studies, in which 100–150 bp fragments were amplified with specific primers to distinguish animal species from *Alheira* sausages and processed fins [39, 40], our results showed that only 133-bp fragments were successfully amplified with template DNA extracted from 15 batches of products. This suggested that the DNA obtained from highly processed products were highly degraded. Hence, specific-PCR combined with DNA mini-barcoding may be a powerful tool for verifying labeling compliance and evaluating the possible existence of fraudulent practices [39, 41–43]. In addition, it is useful for identification of archival specimens and expands the application of DNA barcoding.

It is important to note that more studies concentrated on the distinctions between black-spotted and red-spotted Tokay Geckos. Black-spotted Tokay Geckos are the authentic medical material while red-spotted Tokay Geckos are a substitute [1, 44]. Numerous studies show significant differences between the black-spotted and red-spotted Tokay Geckos based on vocalization analyses, 12S rRNA gene sequences, complete mitochondrial genome and mitochondrial cytochrome *b* gene sequences, nuclear DNA information, karyotypes, and microsatellite loci analyses [45–50]. Furthermore, Qin, Zhang, and Kongbuntad found that *G. gecko* were genetically diverse in relation to their geographical distribution [51–53]. Furthermore, two types of *G. gecko sp.* may have subspecies, despite the fact that the red-spotted Tokay Gecko has been classified as *G. gecko* in Chinese Pharmacopoeia since 2010 [54]. At present, due to limited specimens and comprehensive data, there is still not enough evidence to support the viewpoint that black-spotted Tokay Gecko and red-spotted Tokay Gecko are different species. Specific primers COISF3/COISR3 from this study may be applied to amplify old museum specimens and acquire more information for the revision of the *G. gecko* phylogeny.

The specific primers are sensitive identifiers of samples with and without the Tokay Gecko but fail to recognize admixtures. Pentaplex PCR technology and DNA barcoding techniques, combined with high resolution melting (Bar-HRM method), can detect adulterants in admixtures based on mini-barcoding with universal primers efficiently [55–59]. However, the variable sites of the *COI* sequence of the 16 species in this study were so many that universal primers of mini-barcoding could be developed successfully. Further research is required to propose a suitable region for Bar-HRM identification based on mini-barcoding with universal primers.

## Conclusion

In this study, *G. gecko* and its 15 adulterants were distinguished from each other through DNA barcoding based on *COI* sequences. Two pairs of specific primers COISF2/COISR2 and COISF3/COISR3 of *G. gecko* were designed to amplify 200-bp and 133-bp fragments of *COI* region, both of which were suitable for the identification of *G. gecko* and its adulterants. Importantly, specific primers COISF3/COISR3 could distinguish *G. gecko* from its products. Consequently, the specific DNA mini-barcoding method established from this study is a powerful tool for the identification of *G. gecko* and its adulterants. Therefore, this method could complement the traditional identification methods and lead to the selective identification of *G. gecko* from highly processed products. This study also provides insight into the identification of Chinese patent medicines.

## Supplementary information

**Additional file 1.** Information of *Gekko gecko* and its adulterants, *COI *sequences analysis, figures of amplification of *Gekko gecko* from related products with *COI* universal primers and specific primers. **Table S1.** The information of *Gekko gecko* and its adulterants in this study. **Table S2.** *COI* sequences characteristics and K2P distances of *Gekko gecko* and its adulterants. **Figure S1.** The Neighbor-joining (NJ) tree based on haplotypes of *Gekko gecko* and its adulterants’ *COI* sequences. The bootstrap values (1000 replicates) were showed (≥50%) for each branch. The number of samples producing each haplotype was noted in “( )”. **Figure S2.** Alignment of specific primers COISF2/COISR2 binding regions of *G. gecko* and adulterants. The number of samples producing each haplotype was noted in “( )”. **Figure S3.** Alignment of specific primers COISF3/COISR3 binding regions of *G. gecko* and adulterants. The number of samples producing each haplotype was noted in “( )”. **Figure S4.** Amplification of *Gekko gecko* from related products. **a** Amplification with *COI* universal primer pair LCO1490/HCO2198. **b** Amplification with *COI* specific primer pair COISF2/COISR2. **c** Amplification with *COI* specific primer pair COISF3/COISR3. 1-3: Renshen Gejie Powder, 4-6: Shenge Pingchuan Capsule, 7-9: Gejie Dangshen Syrup, 10-12: Gejie Dingchuan Pill, 13-15: Gejie Dingchuan Capsule, CK: negative control.

## Data Availability

The materials are available from the corresponding author on reasonable reguest.
