# Supplementary material for: Specific DNA mini-barcoding for identification of Gekko gecko and its products
Source: Chin Med. 2020 Sep 29;15:103. doi: 10.1186/s13020-020-00382-2 (PMC7526243; doi:10.1186/s13020-020-00382-2)
Supplement: Supplementary file 1 — Additional file 1. Information of Gekko gecko and its adulterants, COI sequences analysis, figures of amplification of Gekko gecko from related products with COI universal primers and specific primers. Table S1. The information of Gekko gecko and its adulterants in this study. Table S2. COI sequences characteristics and K2P distances of Gekko gecko and its adulterants. Figure S1. The Neighbor-joining (NJ) tree based on haplotypes of Gekko gecko and its adulterants’ COI sequences. The bootstrap values (1000 replicates) were showed (≥50%) for each branch. The number of samples producing each haplotype was noted in “( )”. Figure S2. Alignment of specific primers COISF2/COISR2 binding regions of G. gecko and adulterants. The number of samples producing each haplotype was noted in “( )”. Figure S3. Alignment of specific primers COISF3/COISR3 binding regions of G. gecko and adulterants. The number of samples producing each haplotype was noted in “( )”. Figure S4. Amplification of Gekko gecko from related products. a Amplification with COI universal primer pair LCO1490/HCO2198. b Amplification with COI specific primer pair COISF2/COISR2. c Amplification with COI specific primer pair COISF3/COISR3. 1-3: Renshen Gejie Powder, 4-6: Shenge Pingchuan Capsule, 7-9: Gejie Dangshen Syrup, 10-12: Gejie Dingchuan Pill, 13-15: Gejie Dingchuan Capsule, CK: negative control. [file 13020_2020_382_MOESM1_ESM.docx]

**Supplementary Information**

**Supplementary Table S1.** The information of *Gekko gecko* and its adulterants in this study.

| **Species** | **Total number of sample** | **Sample No.** | **GenBank No.** | **Source** | **Type of sample** |
| --- | --- | --- | --- | --- | --- |
| *Gekko gecko* | 143 | DW001GJ01001-004 | MG257495-498 | Nanjing, Jiangsu | Original animal form farm (Black-spotted Tokay Gecko) |
|  |  | DW001GJ02001-002 | MG257499-500 | National Institute for Food and Drug Control | Crude medicinal material (Red-spotted Tokay Gecko) |
|  |  | DW001GJ03001-020 | MG257501-520 | Anguo herb market | Crude medicinal material (Red-spotted Tokay Gecko) |
|  |  | DW001GJ04001-014 | MG257521-534 | Market (Sichuan) | Crude medicinal material (Red-spotted Tokay Gecko) |
|  |  | DW001GJ05001-029 | MG257535-563 | Market (Sichuan) | Crude medicinal material (Red-spotted Tokay Gecko) |
|  |  | DW001GJ06001-010 | MG257564-573 | Market (Tibet) | Crude medicinal material (Red-spotted Tokay Gecko) |
|  |  | DW001GJ07001-010 | MG257574-583 | Market (Xinjiang Province) | Crude medicinal material (Red-spotted Tokay Gecko) |
|  |  | DW001GJ08001-004 | MG257584-587 | Xinjiang Province | Crude medicinal material (Red-spotted Tokay Gecko) |
|  |  | DW001GJ09001-010 | MG257588-597 | Guangxi Province | Crude medicinal material (Red-spotted Tokay Gecko) |
|  |  | DW001GJ10001-040 | MG257598-637 | Bozhou herb market | Crude medicinal material (Red-spotted Tokay Gecko) |
| *Gekko swinhonis* | 24 | DW002WP01001-010 | MG257638-647 | Anguo herb market | Crude medicinal material |
|  |  | DW002WP02001 | MG257648 | Market (Sichuan Province) | Crude medicinal material |
|  |  | DW002WP03001-013 | MG257649-661 | Renqiu, Hebei | Original animal form farm |
| *Hemidactylus frenatus* | 10 | DW003YW01001-009  DW003YW02001 | MG257662-670 MG257671 | Market (Sichuan Province) | Crude medicinal material |
| *Acanthosaura lepidogaster* | 3 | DW004LJ01001-003 | MG257672-674 | Yunnan Province | Specimen |
| *Calotes kakhienensis* | 3 | DW005BX01001-003 | MG257675-677 | Yunnan Province | Specimen |
| *Calotes versicolor* | 3 | DW006BS01001-002 | MG257678-679 | Hainan Province | Specimen |
|  |  | - | KC875815 | GenBank |  |
| *Laudakia himalayana* | 22 | DW007XS01001-005 | MG257680-684 | Market (Tibet) | Crude medicinal material |
|  |  | DW007XS02001-016 | MG257685-700 | Market (Sichuan Province) | Crude medicinal material |
|  |  | DW007XS03001 | MG257701 | Lasa, Tibet | Specimen |
| *Laudakia sacra* | 3 | DW008LS01001-003 | MG257702-704 | Lasa, Tibet | Specimen |
| *Phrynocephalus axillaris* | 3 | DW009YC01001 | MG257705 | Xinjiang Province | Specimen |
|  |  | - | KC119493,  KF691724 | GenBank |  |
| *Phrynocephalus mystaceus* | 3 | DW010DE01001-002 | MG257706-707 | Xinjiang Province | Crude medicinal material |
|  |  | - | KC578685 | GenBank |  |
| *Phrynocephalus theobaldi* | 3 | DW011XZ01001-002 | MG257708-709 | Lasa, Tibet | Specimen |
|  |  | - | KJ551842 | GenBank |  |
| *Eumeces chinensis* | 9 | DW012SL01001-005 | MG257710-714 | Anguo herb market | Crude medicinal material |
|  |  | DW012SL02001-004 | MG257715-718 | Market (Sichuan Province) | Crude medicinal material |
| *Batrachuperus pinchonii* | 3 | DW013SX01001-003 | MG257719-721 | Market (Sichuan Province) | Crude medicinal material |
| *Cynops orientalis* | 20 | DW014DF01001-010 | MG257722-731 | Huanggang, Hubei | Original animal form farm |
|  |  | DW014DF02001-010 | MG257732-741 | Guangxi Province | Original animal form farm |
| *Paramesotriton chinensis* | 10 | DW015ZG01001-010 | MG257742-751 | Vietnam | Original animal form farm |
| *Tylototriton shanjing* | 12 | DW016HL01001-010 | MG257752-761 | Market (Tibet) | Crude medicinal material |
|  |  | DW016HL02001-002 | MG257762-763 | Yunnan Province | Specimen |

**Supplementary Table S2.** *COI* sequences characteristics and K2P distances of *Gekko gecko* and its adulterants.

| **Species** | **Total number of sample** | **Number of haplotype** | **Sequence**  **length/bp** | **Average GC**  **content/%** | **Intraspecific K2P**  **distance (mean)** | **Interspecific K2P**  **distance (mean)** |
| --- | --- | --- | --- | --- | --- | --- |
| *Gekko gecko* | 143 | 21 | 658 | 48.2 | 0-0.015 (0.005) | 0.273-0.462 (0.341) |
| *Gekko swinhonis* | 24 | 5 | 658 | 45.3 | 0-0.054 (0.008) | 0.263-0.465 (0.310) |
| *Hemidactylus frenatus* | 10 | 5 | 658 | 50.6 | 0-0.079 (0.045) | 0.277-0.452 (0.331) |
| *Acanthosaura lepidogaster* | 3 | 1 | 658 | 46.0 | 0 | 0.312-0.465 (0.434) |
| *Calotes kakhienensis* | 3 | 1 | 658 | 44.4 | 0 | 0.284-0.462 (0.425) |
| *Calotes versicolor* | 3 | 2 | 658 | 45.1 | 0-0.002 (0.001) | 0.295-0.463 (0.420) |
| *Laudakia himalayana* | 22 | 6 | 655 | 44.7 | 0-0.005 (0.001) | 0.033-0.465 (0.416) |
| *Laudakia sacra* | 3 | 1 | 655 | 44.9 | 0 | 0.033-0.463 (0.394) |
| *Phrynocephalus axillaris* | 3 | 1 | 611 | 39.9 | 0 | 0.132-0.431 (0.363) |
| *Phrynocephalus mystaceus* | 3 | 2 | 655 | 41.6 | 0-0.002 (0.001) | 0.147-0.459 (0.408) |
| *Phrynocephalus theobaldi* | 3 | 3 | 655 | 40.2 | 0.002-0.019 (0.012) | 0.132-0.447 (0.372) |
| *Eumeces chinensis* | 9 | 1 | 658 | 45.9 | 0 | 0.252-0.441 (0.318) |
| *Batrachuperus pinchonii* | 3 | 3 | 658 | 39.5 | 0.012-0.046 (0.031) | 0.244-0.437 (0.322) |
| *Cynops orientalis* | 20 | 9 | 658 | 45.5 | 0-0.017 (0.007) | 0.140-0.460 (0.304) |
| *Paramesotriton chinensis* | 10 | 3 | 658 | 44.9 | 0-0.003 (0.001) | 0.140-0.463 (0.312) |
| *Tylototriton shanjing* | 12 | 6 | 658 | 46.2 | 0-0.008 (0.003) | 0.217-0.425 (0.299) |


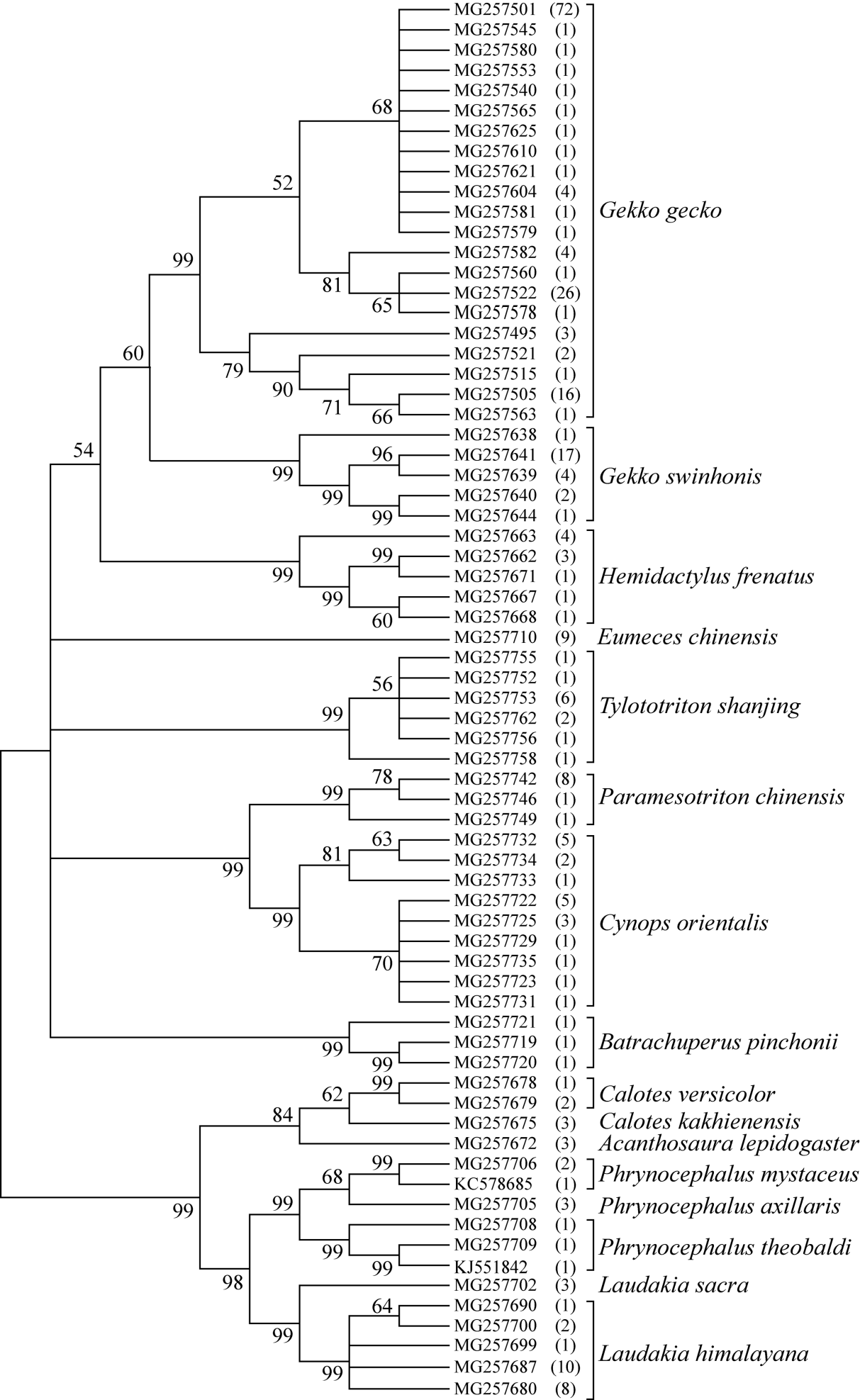


**Supplementary Figure S1.** The Neighbor-joining (NJ) tree based on haplotypes of *Gekko gecko* and its adulterants’ *COI* sequences. The bootstrap values (1000 replicates) were showed (≥50%) for each branch. The number of samples producing each haplotype was noted in “( )”.

**
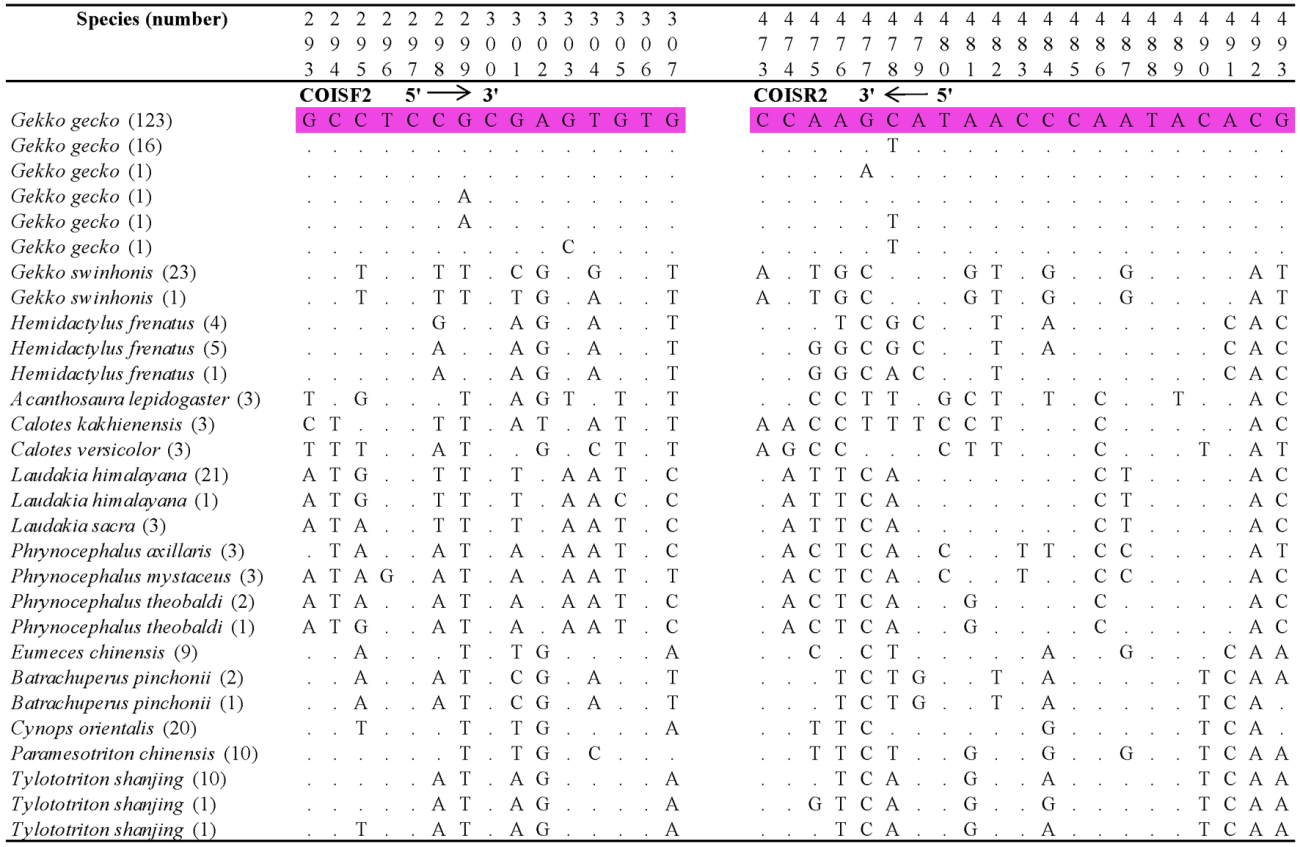
**

**Supplementary Figure S2.** Alignment of specific primers COISF2/COISR2 binding regions of *G. gecko* and adulterants. The number of samples producing each haplotype was noted in “( )”.

**
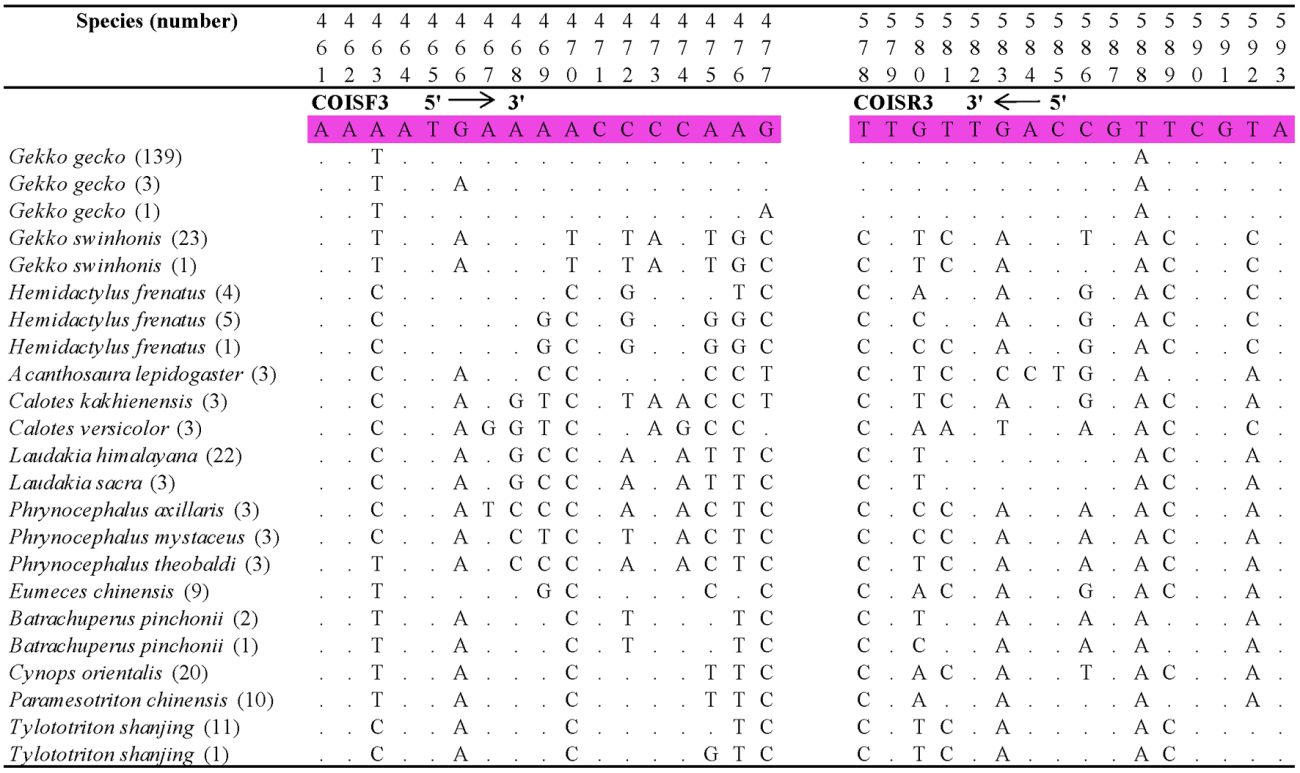
**

**Supplementary Figure S3.** Alignment of specific primers COISF3/COISR3 binding regions of *G. gecko* and adulterants. The number of samples producing each haplotype was noted in “( )”.

**
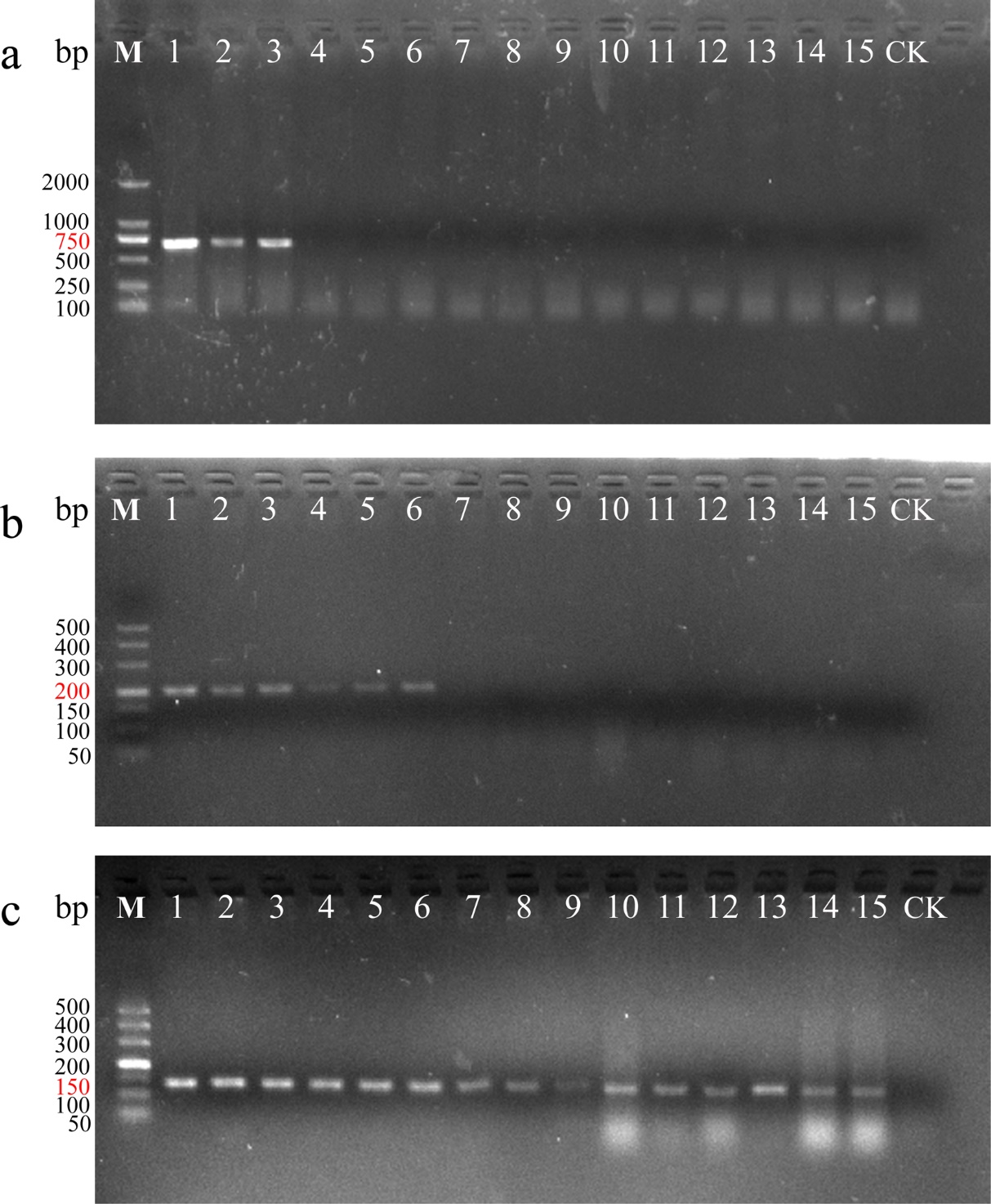
**

**Supplementary Figure S4.** Amplification of *Gekko gecko* from related products. **a** Amplification with *COI* universal primer pair LCO1490/HCO2198. **b** Amplification with *COI* specific primer pair COISF2/COISR2. **c** Amplification with *COI* specific primer pair COISF3/COISR3. 1-3: Renshen Gejie Powder, 4-6: Shenge Pingchuan Capsule, 7-9: Gejie Dangshen Syrup, 10-12: Gejie Dingchuan Pill, 13-15: Gejie Dingchuan Capsule, CK: negative control.
